# Application of proteomics to identify the target molecules involved in *Lonicera japonica*-induced photokilling in human lung cancer CH27 cells

**DOI:** 10.1186/1472-6882-13-244

**Published:** 2013-10-01

**Authors:** Jung C Liao, Wen T Chang, Yu H Lan, Mann J Hour, Hong Z Lee

**Affiliations:** 1 School of Pharmacy, China Medical University, Taichung, Taiwan; 2 School of Chinese Pharmaceutical Sciences and Chinese Medicine Resources, China Medical University, Taichung, Taiwan

**Keywords:** *Lonicera japonica*, Human lung squamous carcinoma CH27 cells, Photocytotoxicity, 2D electrophoresis, Mitochondrial chaperones, Endoplasmic reticulum chaperones

## Abstract

**Background:**

The *Lonicera japonica* has been used as natural and healthy drink for its anti-inflammatory effect and pleasant odor in China and Taiwan.

**Methods:**

2D electrophoresis was used to analyze the proteins involved in photoactivated *Lonicera japonica*-induced CH27 cell apoptosis. The fluorescent dyes MitoTracker Red CMXRos, calcein AM and JC-1 were used to elucidate mitochondrial function. The protein expression was performed by Western blotting. Fluorescent image of endoplasmic reticulum was accomplished by using ER-Tracker Green. This study used fluorescent dye CM-H_2_DCFDA to detect intracellular generation of reactive oxygen species.

**Results:**

The identified proteins can be classified into three major groups, which include proteins involved in mitochondrial function, cytoskeleton-related proteins and proteins associated with endoplasmic reticulum (ER) stress. Photoactivated *Lonicera japonica* caused a significant effect on the mitochondrial function and ER stress in CH27 cells. The reactive oxygen species producing was found to be involved in photoactivated *Lonicera japonica*-induced CH27 cell apoptosis.

**Conclusion:**

Mitochondria and endoplasmic reticulum are the integral targets in photoactivated *Lonicera japonica*-induced CH27 cell apoptosis. We also demonstrated that ethyl acetate fraction of *Lonicera japonica* extracts caused photocytotoxicity in a dose-dependent manner in CH27 cells. This could explain the fact that the ethyl acetate fraction of *Lonicera japonica* extracts may contain compounds which exhibit the photosensitizing activity in CH27 cells.

## Background

The *Lonicera japonica* flower is called Jin Yin Hua in China and Taiwan. Jin Yin Hua is often used in traditional Chinese medicine to treat excess heat conditions such as fevers, skin rashes and sore throat. Therefore, Jin Yin Hua has been used as natural and healthy drink for its anti-inflammatory effect and pleasant odor. Recently, the mechanisms of *Lonicera japonica* in anti-inflammatory and anti-tumor activity have been reported [1,2]. *Lonicera japonica* extracts has been found to be an effective non-steroidal anti-inflammatory drug because it shows preferential inhibition toward cyclooxygenase-2 activity and protein expression [2]. Park et al. [1] demonstrated that the polyphenolic extract isolated from *Lonicera japonica* triggered HepG2 cell death through inhibition of PI3K/Akt and activation of mitogen-activated protein kinases (MAPKs). Photodynamic therapy (PDT) which uses the activation of tumor-localizing photosensitizing agents by visible light is an effective therapy for local malignant tumors. In our previous study, *Lonicera japonica* was found to exhibit a significant photocytotoxicity in human lung squamous carcinoma CH27 cells [3]. We also demonstrated that the promotion of the cytoskeleton-related signaling cascade following rottlerin reduced photoactivated *Lonicera japonica*-induced CH27 cell death [4]. However, the molecular mechanisms underlying the biological effects of photoactivated *Lonicera japonica* still remain unknown. Proteomics is now generally accepted as a method to analyze total protein expression and elucidate cellular processes at the molecular level [5,6]. In this study, proteomics was used to identify the marker proteins that are involved in photoactivated *Lonicera japonica*-induced CH27 cell apoptosis.

Many reports emphasize that heat shock proteins (HSPs), which are synthesized by cells in response to various stress conditions, have a complex role in anti-apoptosis. In general, molecular chaperones can be found in both the cytoplasm and organelles, such as the nucleus, mitochondria and endoplasmic reticulum (ER) in cells. It has been reported that overexpression of HSP60 is sufficient to prevent apoptosis by protecting mitochondrial function in cardiac myocytes [7]. HSP70 and HSP27 inhibit apoptosis through prevention recruitment of procaspase-9 from the apaf-1 apoptosome and inhibition cytochrome *c*-dependent activation of procaspase-9 respectively [8,9]. A number of different models have been proposed to explain that the ER serves as a critical apoptotic control point and has a complex role in apoptosis [10-12]. Endoplasmic reticulum stress, which is induced by the accumulation of misfolded proteins in the endoplasmic reticulum, can initiate cell death under pathological conditions [12]. When cells accumulate ER stresses, ER chaperones can be induced to alleviate protein aggregation and activate the proteosome machinery to degrade misfolded proteins. Furthermore, Feng et al. [10] suggested that induction of ER stress protects gastric cancer cells against apoptosis induced by cisplatin and doxorubicin through activation of p38 MAPK.

Free radicals are a family of molecules, which modulate several important physiological functions including proliferation and apoptosis. Many studies reported that reactive oxygen species (ROS) participated in apoptosis through inducing mitochondrial dysfunction [11,13]. Furthermore, reactive oxygen species production is believed to play a key role in photosensitizer-induced photocytotoxicity. Many investigators have suggested that PDT involves activation of a photosensitizer, which induced singlet oxygen and other reactive oxygen species formation after exposure to light, causing cancer cells to undergo apoptosis or necrosis [14-17].

According to our study of 2D electrophoresis, molecular chaperone proteins involved in mitochondrial function and ER stress, and cytoskeleton-related proteins were participated in photoactivated *Lonicera japonica*-induced CH27 cell death. Molecular chaperone expression, mitochondrial function and endoplasmic reticulum stress served as important apoptotic control point in photoactivated *Lonicera japonica*-induced apoptosis in this study.

## Methods

### Materials

Antipain, aprotinin, dithiothreitol (DTT), ethyleneglycol-bis-(β-aminoethyl ether)-N,N,N’,N’-tetraacetic acid (EGTA), leupeptin, pepstatin, phenylmethylsulfonyl fluoride (PMSF) and Tris were purchased from Sigma Chemical Company (St. Louis, MO, USA). Antibodies to various proteins were obtained from the following sources: β-Actin was from Sigma Chemical Company. PDI and HSP70 were purchased from BD Biosciences (San Diego, CA, USA). HSP60 was purchased from Calbiochem (San Diego, CA, USA). 150 kDa Oxygen-regulated protein (ORP150) was purchased from IBL (Japan). Caspase-4 was purchased from Abcam (Cambridge, MA, USA). MitoTracker Red CMXRos and ER-Tracker Green reagent were from Molecular Probes (Eugene, OR, USA).

### Preparation of *Lonicera japonica*

The botanical origin of *Lonicera japonica* was identified by Dr. Chao-Lin Kuo (School of Chinese Pharmaceutical Sciences and Chinese Medicine Resources, China Medical University, Taichung, Taiwan). The voucher specimen (*Lonicera japonica:* CMU LJ 0614) was deposited in School of Chinese Pharmaceutical Sciences and Chinese Medicine Resources, China Medical University, Taichung, Taiwan. The air-dried plants of *Lonicera japonica* (200 g) were soaked three times with 1 L of 95% ethanol at room temperature for 3 days. The extracts were filtered. The filtrates were collected and then concentrated under reduced pressure at 40°C. The yield of dry extract of *Lonicera japonica* was about 11%.

### Cell culture

CH27 cells were grown in monolayer culture in Dulbecco’s modified Eagle’s medium (DMEM; Life Technologies, Rockville, MD, USA) containing 5% FBS (HyClone, Logan, UT, USA), 100 U/ml penicillin, 100 μg/ml streptomycin (Gibco BRL, Rockville, MD, USA) and 2 mM glutamine (Merck, Darmstadt, Germany) at 37°C in a humidified atmosphere comprised of 95% air and 5% CO_2_. When CH27 cells were treated with *Lonicera japonica*, the culture medium containing 1% FBS was used. All data presented in this study are from at least 3 independent experiments.

### Light source

The irradiation source was a set of fluorescent lamp (2 × 20 W; China Electric MFG Corporation, Taiwan, R.O.C.) located in a made-to-measure box. The wavelength of the fluorescence lamp was in the range of 400-700 nm. The intensity of light was measured as Lux and Lux was converted to light dose (J/cm^2^). The cells were irradiated at 40 W for 30 min, corresponding to 0.8 J/cm^2^ light dose.

### Protein preparation

Cells were seeded at a density of 1.7 × 10^6^ cells onto 10-cm dish 48 h before being treated with drugs. CH27 cells were incubated with 0.1% dimethylsulfoxide (DMSO) or 100 μg/ml *Lonicera japonica* extracts and then irradiated with 0.8 J/cm^2^ fluence dose. After irradiation, adherent and floating cells were collected and washed twice in ice-cold phosphate-buffered saline (PBS). Cell pellets were resuspended in cell lysis buffer (50 mM Tris-HCl, pH 7.5, 150 mM sodium chloride, 1% Nonidet P-40, 0.25% sodiumdeoxycholate, 1 mM EGTA, 1 mM DTT, 1 mM PMSF, 1 mM sodium orthovanadate, 1 mM sodium fluoride, 5 μg/ml aprotinin, 5 μg/ml leupeptin and 5 μg/ml antipain) for 30 min at 4°C. Lysates were clarified by centrifugation at 13,000 rpm for 30 min at 4°C. The resulting supernatant was collected, aliquoted (150 μg/tube for 2D electrophoresis and 50 μg/tube for Western blot) and stored at −80°C until assay. The protein concentrations were estimated with the Bradford method.

### Two-dimensional gel electrophoresis

The proteins (150 μg) were dissolved in a rehydration buffer (9.8 M urea, 0.5% CHAPS, 10 mM DTT, 0.2% Biolytes and a trace of bromophenol blue) to a final volume of 125 μl. The samples were added to the 7-cm IPG strips (pH 4-7, linear, Readystrip; BioRad, Hercules, CA), which were rehydrated for 12 h. After rehydration, the strips were focused for 60,000 Vh, starting at 250 V and gradually raising the voltage to 10,000 V. Once the IEF was completed, the strips were equilibrated in 6 M urea containing 2% SDS, 0.375 M Tris (pH 8.8), 20% glycerol and 130 mM DTT. The 2D electrophoresis was performed using 12% sodium dodecyl sulfate-polyacrylamide gel electrophoresis (SDS-PAGE).

### Silver staining of proteins

Gels were fixed in 50% methanol (v/v) and 12% acetic acid (v/v) for 2 h, and then washed 3 times in 50% ethanol (v/v). The duration of each wash was 20 min. Gels were then incubated in a 0.02% sodium thiosulfate solution (w/v) for 1 min, followed by four 1-min washes in water. Gels were then placed in a solution composed of 0.2% silver nitrate (w/v) and 0.075% (v/v) formaldehyde for a period of 20 min, followed by three 1-min washes in water. Gels were then developed in a 6% sodium carbonate (w/v), 0.005% formaldehyde (v/v) and 0.004% sodium thiosulfate (w/v) solution until the protein spots were visualized. A 1% acetic acid solution was added to stop the staining reactions.

### NanoLC-MS/MS analysis and database searches

NanoLC-MS/MS analysis was performed on an integrated nanoLC-MS/MS system (QSTAR XL) comprising a LC Packings NanoLC system with an autosampler and a QSTAR XL Q-Tof mass spectrometer (Applied Biosystems, Foster City, CA) fitted with nanoLC sprayer. Mass analysis was carried out according to the Analyst QS software (Applied Biosystems). The proteins were identified by searching in SWISS-PROT and NCBI database using the Mascot program with the following parameters: peptide mass tolerance, 50 ppm; MS/MS ion mass tolerance, 0.25 Da; and allow up to one missed cleavage. Only significant hits as defined by Mascot probability analysis will be considered initially.

### Measurement of mitochondrial function

CH27 cells were incubated with 0.1% DMSO or *Lonicera japonica* for 4 h and then irradiated with 0.8 J/cm^2^ light dose. Mitochondrial activity, the opening of mitochondrial permeability transition (MPT) pore and mitochondrial membrane potential (MMP) were measured as previously described [4,11]. To detect mitochondrial activity, cells were incubated for 30 min at 37°C with 100 nM MitoTracker Red CMXRos and observed by fluorescent microscope (Olympus IX 70). To measure the opening of MPT pore, the fluorescence intensity of calcein was measured with FACSCanto flow cytometer (excitation, 488 nm; emission, 530 nm) and analysed using ModFit LT 3.0 Software (Verity Software House, Topsham, ME, USA). MMP was determined by flow cytometry analysis of JC-1 (5,5′,6,6′-tetrachloro-1,1′,3,3′-tetraethylbenzimidazolocarbocyanine iodide)-stained cells.

### Western blot analysis

Samples were separated by various indicated concentrations of SDS-PAGE. The SDS-separated proteins were equilibrated in transfer buffer (50 mM Tris-HCl, pH 9.0-9.4, 40 mM glycine, 0.375% SDS and 20% methanol) and electrotransferred to Immobilon-P transfer membranes (Millipore Corporation, Bedford, MA). The blot was blocked with a solution containing 5% nonfat dry milk in TBST for 1 h, washed and incubated with antibodies to β-actin (1:5000 [Sigma], the detection of β-actin was used as an internal control in the data of Western blotting analysis), ORP150 (1:100), PDI (1:250), HSP60 (1:7000), HSP70 (1:1000) and caspase-4 (1:12,000). Secondary antibody consisted of a 1:20,000 dilution of horseradish peroxidase (HRP)-conjugated goat anti-mouse IgG (for β-actin, ORP150, PDI, HSP60 and HSP70) and HRP-conjugated goat anti-rabbit IgG (for caspase-4). The enhanced chemiluminescent (NEN Life Science Products, Boston, MA) detection system was used for immunoblot protein detection.

### Fluorescent imaging of endoplasmic reticulum (ER)

Fluorescent imaging of endoplasmic reticulum was accomplished using ER-Tracker Green (glibenclamide-BODIPY FL) and used as directed by the manufacturer. CH27 cells were seeded onto 12-well plate 48 h before being treated with *Lonicera japonica* and light. After irradiation, cells were incubated for 30 min at 37°C with 1 μM of ER-Tracker. The cells were then washed with PBS and examined by fluorescence microscopy (300×).

### Measurement of reactive oxygen species production

This study used 5-(and-6)-chloromethyl-20,70-dichlorodihydrofluorescein diacetate (CM-H_2_DCFDA, Molecular Probes) to detect intracellular generation of reactive oxygen species. CH27 cells were loaded with 5 μM CM-H_2_DCFDA for 40 min in the dark. During loading, the acetate groups on CM-H_2_DCFDA were removed by intracellular esterase, trapping the probe inside the cells. Cells loaded with CM-H_2_DCF were treated with various indicated concentrations of *Lonicera japonica* and then irradiated with 0.8 J/cm^2^ light dose. After irradiation, production of reactive oxygen species can be measured by changes in fluorescence due to intracellular production of CM-DCF caused by oxidation of CM-H_2_DCF. CM-DCF fluorescence was measured using a fluorometer (Thermo Scientific Fluoroskan Ascent FL; Helsinki, Finland) at an excitation wavelength of 480 nm and an emission wavelength of 520 nm.

### Fractionation of the *Lonicera japonica* extracts

The alcoholic extracts of *Lonicera japonica* was suspended in water and partitioned successively with ethyl acetate (EtOAc) and *n*-butanol (BuOH). Each extract was evaporated to dryness under reduced pressure to yield water, ethyl acetate and *n*-butanol fractions.

### Mitochondrial Reductase activity

Cells were seeded at a density of 1 × 10^5^ cells per well onto 12-well plate 48 h before being treated with drugs. The cells were incubated with 0.1% DMSO or partitioned fractions of *Lonicera japonica* extracts and then irradiated with the light dose of 0.8 J/cm^2^. After irradiation, the cells were washed with PBS. Cellular mitochondrial reductase activity of live CH27 cells was determined by measuring the reduction of 3-(4,5-dimethylthiazol-2-yl)-2,5-diphenyltetrazolium bromide (MTT). Cells were incubated with MTT (2.4 × 10^-4^ M) for 1 h at 37°C. After solubilization in dimethylsulfoxide, absorbance was measured at 550 nm.

### Statistical analysis

All experiments were carried out at least three independent experiments. Each sample was tested in triplicate. Standard statistical methods based on Student’s *t* test and regression analysis were used. The results are expressed as percentage ± S.D. of control.

## Results

### Identification of differentially expressed proteins by 2D gel

**Figure 1:**
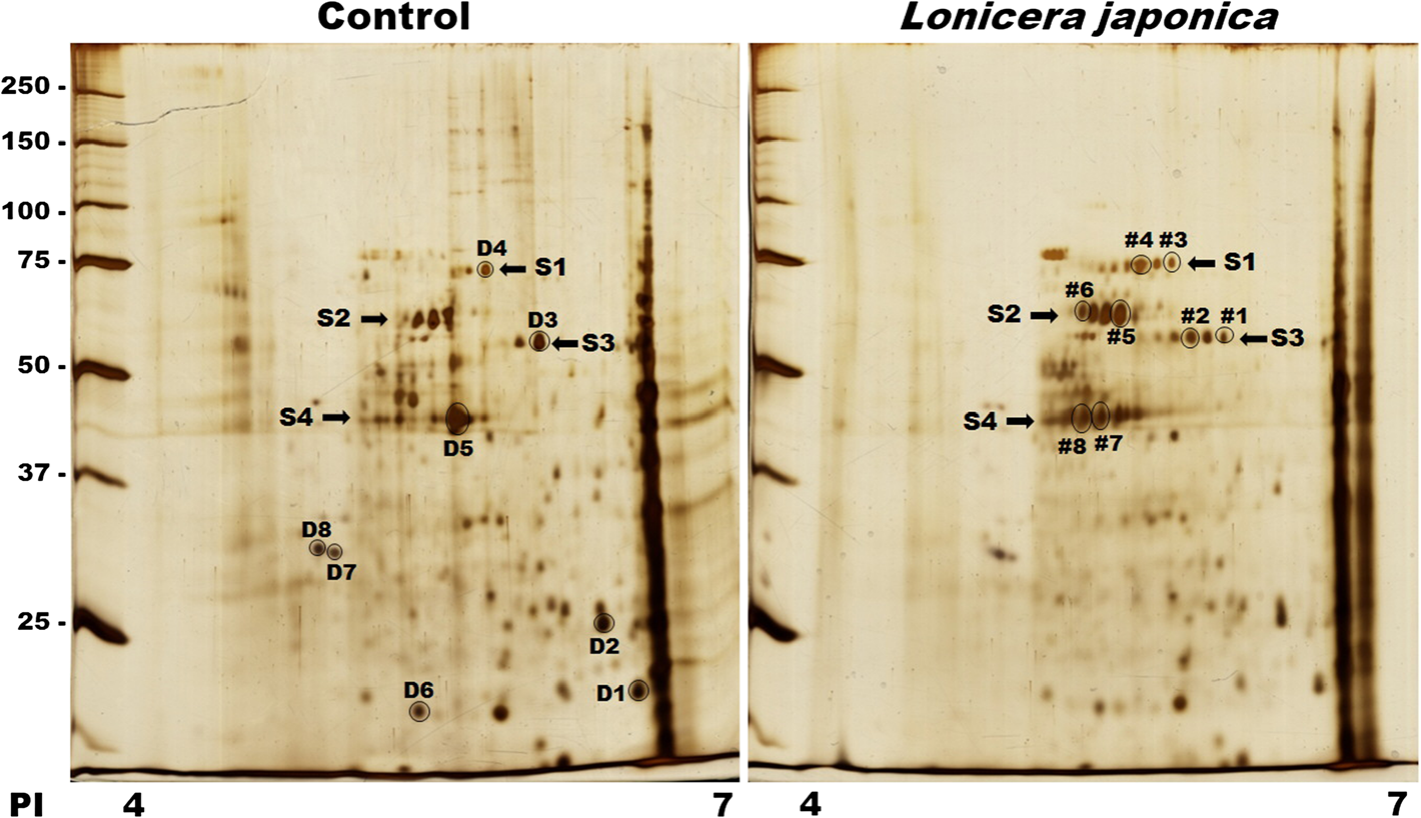
**Two-dimensional electrophoresis maps of control and *Lonicera japonica*-photosensitized CH27 cells.** Cells were incubated with 0.1% DMSO or 100 μg/ml *Lonicera japonica* extracts for 4 h and then irradiated with 0.8 J/cm^2^ fluence dose. Proteins were separated on a pH 4-7 IPG-strip (7 cm) in the first dimension and on a 12% SDS-polyacrylamide gel in the second dimension. Staining of the protein spots was accomplished by silver nitrate. Results are representative of three independent experiments.

The major purpose of this study was to investigate the change in protein level during *Lonicera japonica*-induced photocytotoxicity of CH27 cells. Therefore, this study examine the difference between control cell-loaded and the photoactivated *Lonicera japonica*-treated cell loaded gels in the pH range of 4.75-6.5, using a 4-7 pH range IPG strip in the first dimension. Therefore, those proteins with pI value less than 4 and more than 7 would be missed. The control cells were treated with 0.1% DMSO and then irradiated with 0.8 J/cm^2^ fluence dose. In this study, light alone did not affect cell survival and protein expression (data not shown). After being incubated with 100 μg/ml *Lonicera japonica* for 4 h and 0.8 J/cm^2^ irradiation, many protein spots were found varying in intensity between the control cell-loaded and the photoactivated *Lonicera japonica*-treated cell-loaded gels (Figure 1). Photoactivated *Lonicera japonica* triggered significant decreases in the protein expression of spots D1-D8 (Figure 1). In *Lonicera japonica*-photosensitized cells, the protein expression of spots #1-#8 is more intense than those in the control cells (Figure 1). These altered protein spots were identified by mass spectrometry and Mascot search. As shown in Table 1, the identified proteins can be classified into three major groups, which include proteins involved in mitochondrial function (DJ-1, ATP synthase, heat shock protein 70 and chaperonin, also known as heat shock protein 60), cytoskeleton-related proteins (heat shock protein 27, tropomyosin and actin cytoplasmic 1) and proteins associated with ER stress (protein disulfide isomerase). It is interesting to note that four spot families in the horizontal direction of the 2D gel were found to markedly change after treatment with 100 μg/ml *Lonicera japonica* for 4 h and 0.8 J/cm^2^ light dose (Figure 1). These altered protein spot families were identified as protein disulfide isomerase, heat shock protein 70, chaperonin and actin cytoplasmic 1. These protein spot families are identical in molecular weight but different in pI values. We found six 73-kDa heat shock protein 70 with pI values of 5.23-5.60 (Figure 1; arrow S1), four 61-kDa chaperonin with pI values of 5.15-5.34 (Figure 1; arrow S2), five 57-kDa protein disulfide isomerase with pI values of 5.54-5.88 (Figure 1; arrow S3) and eight 42-kDa actin cytoplasmic 1 proteins with pI values of 4.97-5.43 (Figure 1; arrow S4) in the horizontal direction of the 2D gel. It has been indicated that the single spots of the complex pattern were probably due to post-translational modifications of one particular protein. Our results suggested that photoactivated *Lonicera japonica*-induced changes in protein expression of many proteins include the pattern of their post-translational modifications in CH27 cells.

**Table 1:** MS/MS analysis and characteristics of the analyzed spots

**Spot Protein**	**Accession no.**	**Calculated pI**	**Theoretical pI/Mw (Da)**	**Score**	**Sequence coverage (%)**	**Changed fold (% control)**^**1**^
D1 DJ-1 protein	NP_009193	6.33	6.32 / 19891.05	88	25	40 ± 4
D2 Heat shock protein 27	AAA62175	6.16	5.98 / 22782.52	273	43	46 ± 5
D3 Protein disulfide isomerase	CAA89996	5.85	5.98 / 56782.39	766	40	48 ± 9
D4 Heat shock 70 kDa protein 9	AAH00478	5.59	5.87 / 73680.50	508	28	49 ± 7
D5 Actin, cytoplasmic 1	NP_001092	5.42	5.29 / 41736.73	896	43	41 ± 5
D6 ATP synthase	NP_006347	5.21	5.21 / 18491.21	147	34	48 ± 3
D7 Tropomyosin 3 isoform 2	NP_705935	4.81	4.68 / 32818.79	283	29	45 ± 11
D8 Tropomyosin 3 isoform 4	NP_001036816	4.73	4.73 / 28870.36	283	29	79 ± 5
#1 Protein disulfide isomerase	CAA89996	5.88	5.98 / 56782.39	423	32	383 ± 26
#2 Protein disulfide isomerase	CAA89996	5.70	5.98 / 56782.39	494	32	143 ± 21
#3 Heat shock 70 kDa protein 9B precursor variant	BAD96478	5.60	5.87 / 73680.50	405	25	1594 ± 115
#4 Heat shock 70 kDa protein 8 isoform 1	NP_006588	5.43	5.61 / 53499.58	540	28	366 ± 62
#5 Chaperonin	NP_002147	5.34	5.70 / 61054.64	760	46	145 ± 25
#6 Chaperonin	NP_002147	5.15	5.70 / 61054.64	600	31	386 ± 51
#7 Actin, cytoplasmic 1	NP_001092	5.25	5.29 / 41736.73	336	37	266 ± 10
#8 Actin, cytoplasmic 1	NP_001092	5.15	5.29 / 41736.73	262	39	376 ± 43

^1^The expression of protein spots was quantified by AlphaEase image software. All protein changed folds are expressed as the mean percentage of the relative control ± S.D. from three independent experiments.

### Effects of photoactivated *Lonicera japonica* on mitochondrial function in CH27 cells

**Figure 2:**
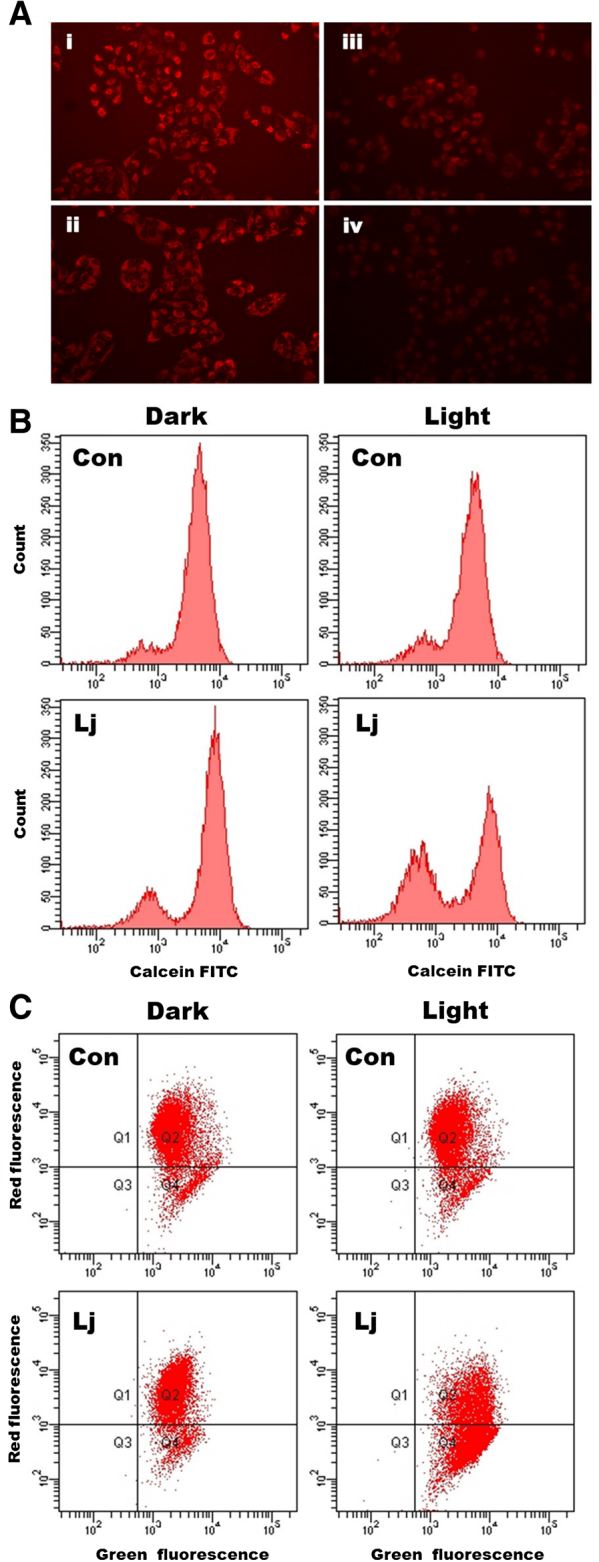
**Effects of photoactivated *Lonicera japonica *extract on mitochondrial function in CH27 cells. (A)** Effects of photoactivated *Lonicera japonica* extracts on the activity of mitochondria in CH27 cells. CH27 cells were incubated with 0.1% DMSO (i) or with 50 (ii), 100 (iii) or 150 (iv) μg/ml *Lonicera japonica* for 4 h and then irradiated with 0.8 J/cm^2^ fluence dose. After treatment, cells were incubated with 100 nM MitoTracker Red CMXRos for 30 min. The specimens were observed by fluorescence microscopy (300×). **(B)** The effect of photoactivated *Lonicera japonica* extracts on opening of mitochondrial permeability transition (MPT) pore in CH27 cells. Cells were incubated with 0.1% DMSO (Con) or 100 μg/mL *Lonicera japonica* extracts (Lj) for 4 h and then irradiated with 0.8 J/cm^2^ fluence dose. Before treatment with *Lonicera japonica* extracts and light, cells were loaded with 1 μM calcein AM for 30 min in DMEM medium containing 1 mM CoCl_2_. After irradiation, the cells were harvested and then analyzed by flow cytometry for loss of fluorescence intensity due to efflux of the dye. In light-shield condition (dark), cells were incubated with 0.1% DMSO (Con) or 100 μg/ml *Lonicera japonica* extracts (Lj) for 5 h. **(C)** The fluorescent cation dye JC-1 was used to determine the mitochondrial membrane potential. Cells were incubated with 0.1% DMSO (Con) or 100 μg/mL *Lonicera japonica* extracts (Lj) for 4 h and then irradiated with 0.8 J/cm^2^ fluence dose. After irradiation, the cells were harvested and stained with 2 μM JC-1 for 15 min. The mitochondrial depolarization patterns of the CH27 cells were measured by flow cytometry. In light-shield condition (dark), cells were incubated with 0.1% DMSO (Con) or 100 μg/ml *Lonicera japonica* extracts (Lj) for 5 h. All results are representative of three independent experiments.

**Figure 3:**
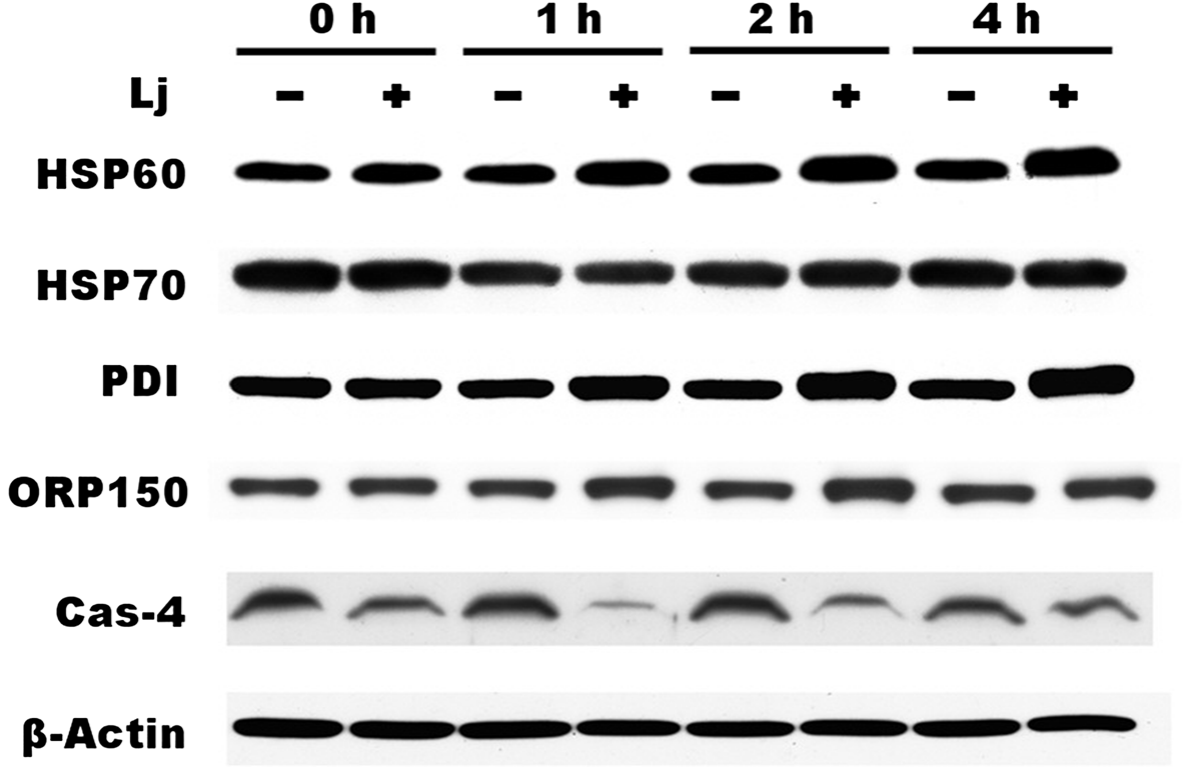
**Effects of photoactivated *Lonicera japonica *extracts on the protein expression of chaperones in CH27 cells.** Cells were incubated with 0.1% DMSO or 100 μg/mL *Lonicera japonica* extracts for 0, 1, 2 and 4 h and then irradiated with 0.8 J/cm^2^ fluence dose. Cell lysates were analyzed by 5% (ORP150), 9% (HSP60 and HSP70), 10% (PDI), 12% (β-actin) and 14% (caspase-4) SDS-PAGE, and then probed with primary antibodies as described in Section 2. −: control cells; +: photoactivated *Lonicera japonica*-treated cells. Results are representative of three independent experiments.

In the results of 2D electrophoresis, photoactivated *Lonicera japonica*-treated samples had a significant change in the expression of DJ-1, ATP synthase, heat shock protein 70 and chaperonin which are involved in mitochondrial function. To examine whether the activity of mitochondria was injured by photoactivated *Lonicera japonica* in CH27 cells, MitoTracker Red CMXRos dye was used in this study. A bright red fluorescence was observed on numerous and dot-like structures in control-treated cells’ cytoplasm (Figure 2A). In photoactivated *Lonicera japonica*-treated cells, the red fluorescence was significantly decreased in a dose-dependent manner (Figure 2A). This result suggested that the capability for MitoTracker Red CMXRos uptake by mitochondria in control cells is higher compared to those in *Lonicera japonica* photosensitized cells. To confirm the possible role of MPT pore in the process of *Lonicera japonica*-induced photocytoxicity, we measured the opening of mitochondrial permeability transition (MPT) pore in intact cells by flow cytometry. As shown in Figure 2B, treatment with 100 μg/ml *Lonicera japonica* extracts for 4 h and then 0.8 J/cm^2^ fluence dose resulted in a decrease in calcein fluorescent intensity because of the opening of MPT pores. However, treatment with 100 μg/ml *Lonicera japonica* extracts for 4 h had no effect on the calcein fluorescent intensity of CH27 cells in the dark (Figure 2B). Because the opening of MPT pore is accompanied by a decrease in mitochondrial membrane potential (MMP), this study examined the photodynamic effect of *Lonicera japonica* on MMP in CH27 cells. After cells were treated with *Lonicera japonica* extracts and irradiation, a remarkable attenuation of MMP occurred compared to the control cells or the cells treated with 100 μg/ml *Lonicera japonica* extracts in the dark (Figure 2C). These results clearly demonstrated that mitochondrial function is severely impaired by photoactivated *Lonicera japonica* in CH27 cells. To further elucidate whether the protein expression of HSP60 (chaperonin) and HSP70 is involved in photoactivated *Lonicera japonica*-induced CH27 cell apoptosis, this study examined the regulation of HSP60 and HSP70 levels by Western blotting analysis. Exposure of CH27 cells to 100 μg/ml *Lonicera japonica* for 2 or 4 h and 0.8 J/cm^2^ irradiation resulted in increases in protein level of HSP60 (Figure 3). However, photoactivated *Lonicera japonica* had no effect on the protein expression of HSP70 (Figure 3).

### ER stress was involved in photoactivated *Lonicera japonica*-induced CH27 cell death

**Figure 4:**
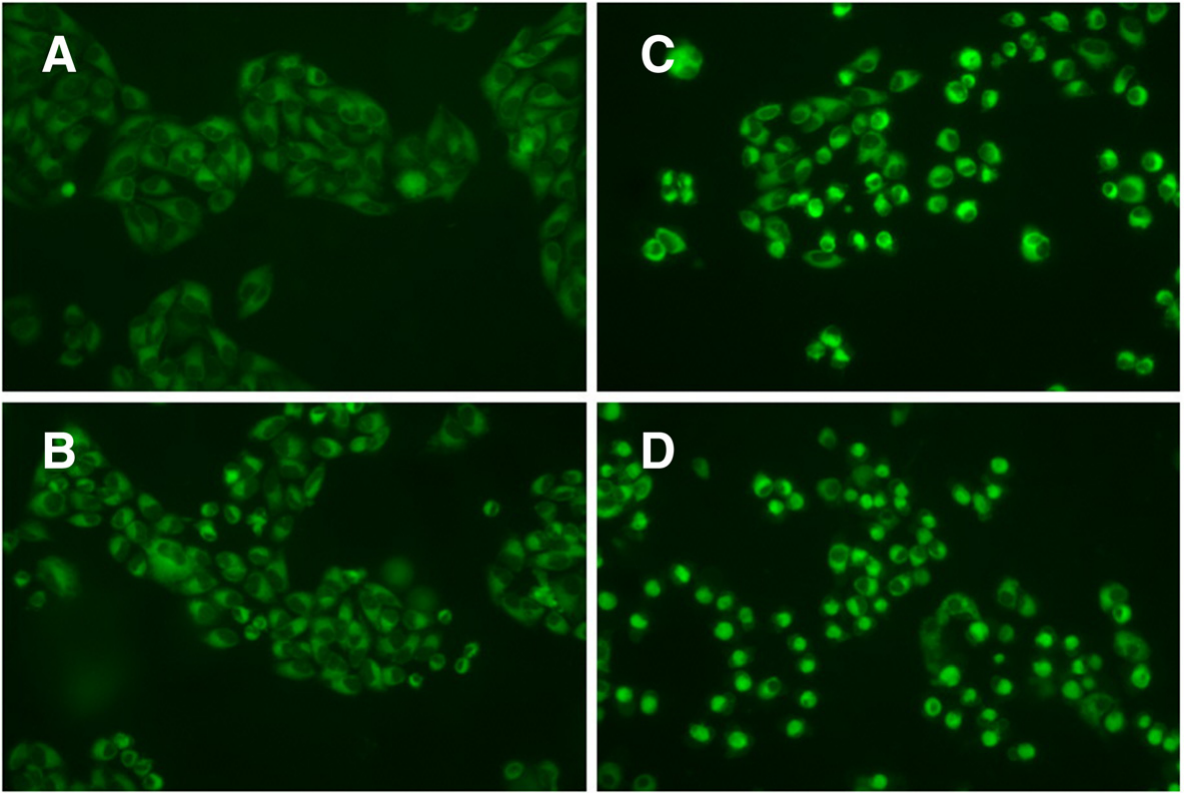
**Effects of photoactivated *Lonicera japonica *on the distribution of endoplasmic reticulum in CH27 cells.** CH27 cells were incubated with 0.1% DMSO **(A)** or with 50 **(B)**, 100 **(C)** or 150 **(D)** μg/ml *Lonicera japonica* for 4 h and then irradiated with 0.8 J/cm^2^ fluence dose. After irradiation, cells were incubated with ER-Tracker and then examined by fluorescence microscopy (300×). Results are representative of three independent experiments.

According to the results of 2D elecrtophoresis, photoactivated *Lonicera japonica* induced a significant change in the protein expression of protein disulfide isomerase (PDI). PDI, a molecular chaperone in the endoplasmic reticulum (ER), catalyzes the formation and breakage of disulfide bonds between cysteine residues within proteins as they fold. To further elucidate whether ER is a target in the photoactivated *Lonicera japonica* (100 μg/ml)-induced CH27 cell death, the protein expression of ER-related molecular chaperones, such as PDI, 150 kDa oxygen-regulated protein (ORP150) and caspase-4, was examined by Western blotting analysis in this study. Exposure of CH27 cells to 100 μg/ml *Lonicera japonica* for 1, 2 or 4 h and 0.8 J/cm^2^ irradiation resulted in increases in protein level of ORP150 and PDI, but caspase-4 level decreased after 2 h (Figure 3). Since ORP150 and PDI are the markers of ER stress, we further investigated the role of ER stress in the photoactivated *Lonicera japonica*-induced apoptosis. In order to demonstrate the role that ER stress plays in the photoactivated *Lonicera japonica*-induced apoptosis, CH27 cells were incubated with an endoplasmic reticulum marker, ER-Tracker™ Green reagent. In control cells, the ER-tracker fluorescence is mostly found in the cytoplasm, consistent with endoplasmic reticulum localization (Figure 4A). As shown in Figure 4, cells exhibited a heterogeneous distribution of the fluorescence after treatment with 100 or 150 μg/ml *Lonicera japonica* for 4 h and 0.8 J/cm^2^ irradiation. Based on the above data, we demonstrated that ER stress was involved in the photoactivated *Lonicera japonica*-induced CH27 cell apoptosis.

### Photoactivated *Lonicera japonica* induced reactive oxygen species (ROS) generation in CH27 cells

**Figure 5:**
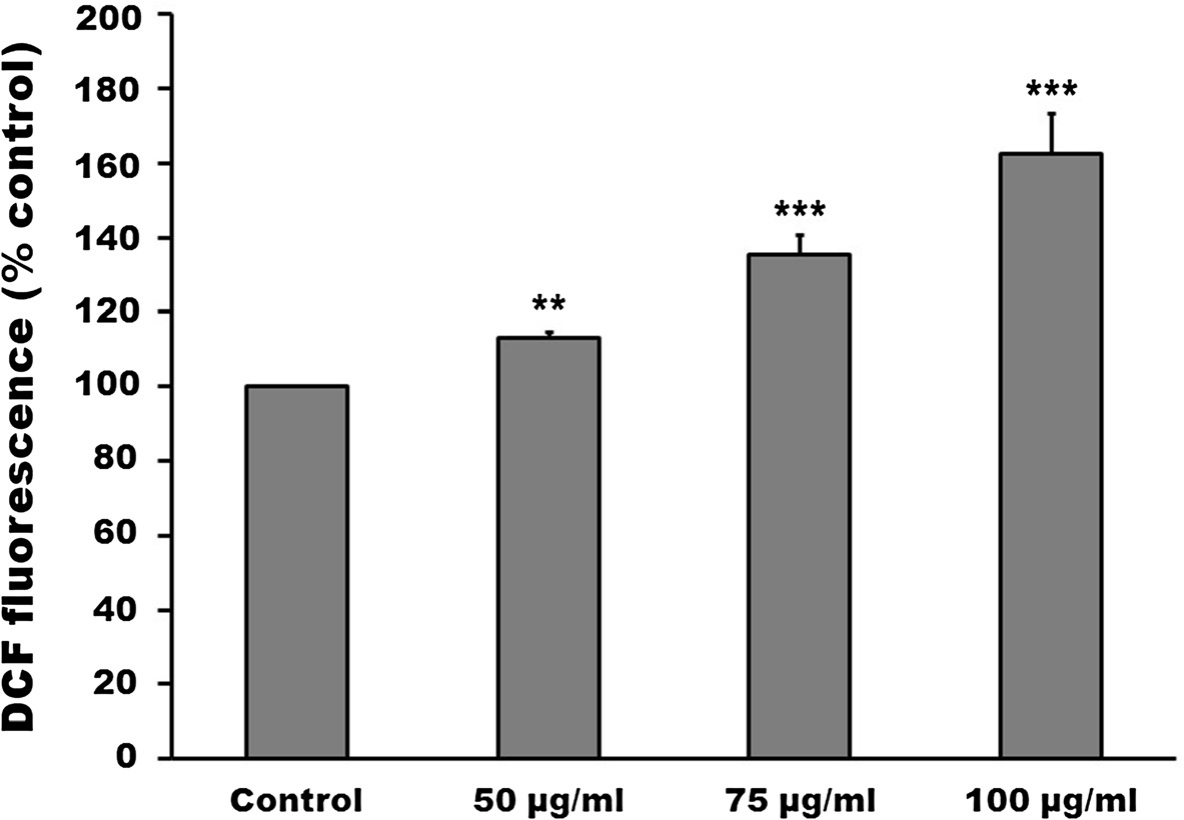
**Effects of photoactivated *Lonicera japonica *on reactive oxygen species (ROS) production in CH27 cells.** Cells were loaded with 5-H_2_DCFDA (5 μM) for 30 min in the dark, washed and then treated with 0.1% DMSO or with 50, 75 or 100 μg/ml *Lonicera japonica* for 2 h and then irradiated with 0.8 J/cm^2^ fluence dose. After irradiation, ROS fluorescence was measured using a multiwell plate reader at an excitation wavelength of 485 nm and an emission wavelength of 538 nm. All results are expressed as the mean percentage of control ± S.D. of triplicate determinations from four independent experiments. ^**^P < 0.01, ^***^P < 0.001 compared to the corresponding control values.

It is well-known that reactive oxygen species participate in apoptosis by inducing the mitochondrial dysfunction and ER stress. In addition, a variety of ROS is produced including singlet molecular oxygen (^1^O_2_, produced *via* type II mechanism) and superoxide anions (type I mechanism) by photosensitizer during the photodynamic process. In order to demonstrate the role that ROS plays in *Lonicera japonica*-induced photocytotoxicity, intracellular ROS generation was examined by using an oxidant sensitive fluorescent probe, CM-H_2_DCFDA. The results showed that treatment with 100 μg/ml *Lonicera japonica* for 2 h and 0.8 J/cm^2^ light dose resulted in significant increases in the intensity of the DCF signal as compared with those in the control (Figure 5). In this study, it was confirmed whether the production of ROS was caused only by irradiation. The amount of ROS induced by 100 μg/ml *Lonicera japonica* in the absence of a light source (without irradiation and dark conditions) was investigated. When CH27 cells were incubated with 0.1% DMSO or 100 μg/ml *Lonicera japonica* for 3 h in the dark, there were no differences in DCF fluorescence intensity between treated and control groups (data not shown).

### The phototoxicity of partitioned fractions of *Lonicera japonica* extracts

**Figure 6:**
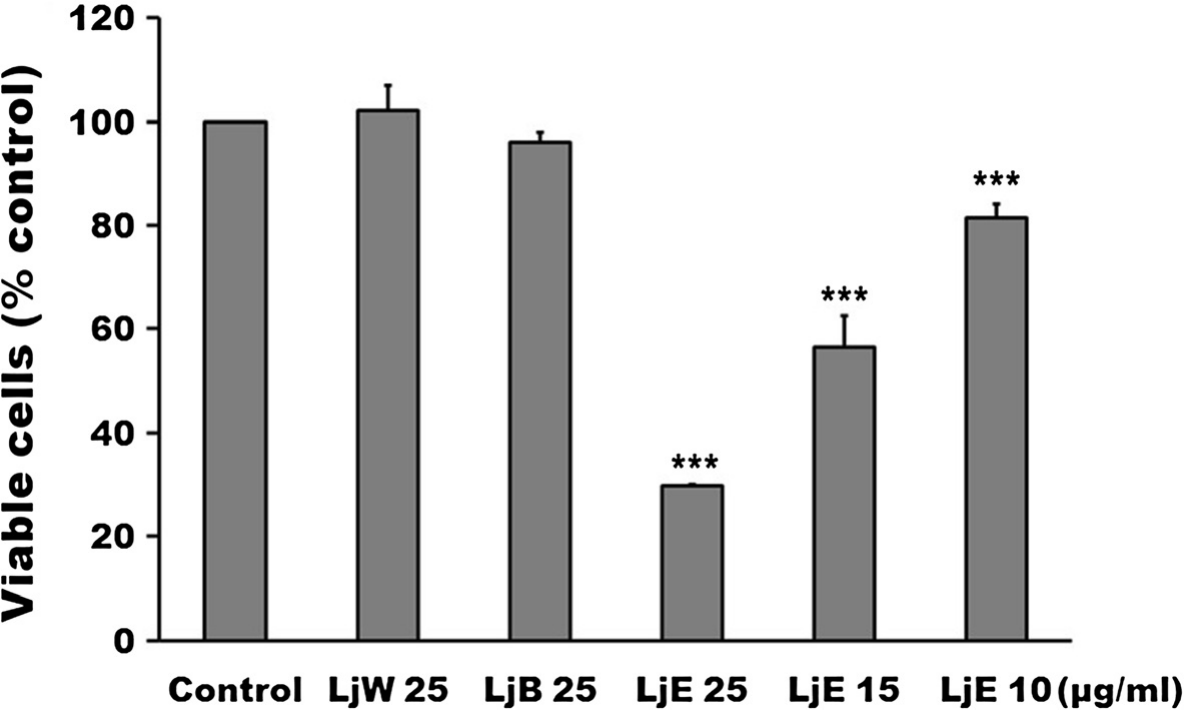
**The photocytotoxicity of partitioned fractions of *Lonicera japonica *extracts of CH27 cells.** Cells were incubated with various concentrations of ethyl acetate fraction of the *Lonicera japonica* extracts or with 25 μg/ml partitioned fractions of *Lonicera japonica* extracts for 4 h and then irradiated with 0.8 J/cm^2^ fluence dose. The viable cells were measured by MTT assay. LjW: water fraction of the *Lonicera japonica* extracts; LjB: *n*-butanol fraction of the *Lonicera japonica* extracts; LjE: ethyl acetate fraction of the *Lonicera japonica* extracts. Results are expressed as the mean percentage of control ± SD. ^***^P < 0.001 compared to the control values.

The present study served to evaluate the potent photokilling fraction of ethanol extracts of *Lonicera japonica.* The alcoholic extracts of *Lonicera japonica* was suspended in water and successively partitioned with ethyl acetate and *n*-butanol. The photocytotoxicity of partitioned fractions (water, ethyl acetate and *n*-butanol) of the *Lonicera japonica* extracts is shown in Figure 6. Results show that the ethyl acetate fraction of the *Lonicera japonica* extracts caused a significant photocytotoxicity in a dose-dependent manner in CH27 cells. The water and *n*-butanol fractions did not show any photokilling effect at 25 μg/ml and 0.8 J/cm^2^ light dose (Figure 6).

## Discussion

In the result of 2D gel electrophoresis, many protein spots were found varying in intensity between the control cell-loaded and the photoactivated *Lonicera japonica*-treated cell-loaded gels. These altered proteins were characterized by mass spectrometry. The identified proteins can be classified into three major groups, which include proteins involved in mitochondrial function (DJ-1, ATP synthase, heat shock protein 70 and chaperonin, also known as heat shock protein 60), proteins associated with ER stress (protein disulfide isomerase) and cytoskeleton-related proteins (heat shock protein 27, tropomyosin and actin cytoplasmic 1). In our previous study, cytoskeleton-related proteins, p38 signaling pathway, have been reported to be involved in photoactivated *Lonicera japonica*-induced CH27 cell apoptosis [3].

Since DJ-1, ATP synthase, HSP60 and HSP70 are mitochondrial protein, we hypothesized that photoactivated *Lonicera japonica* induces CH27 cell apoptosis in association with regulation of mitochondrial function. The MPT pore is characterized by opening of the permeability transition pore in the inner mitochondrial membrane, which results in an increase in permeability of this membrane to protons, ions and small-molecular weight solutes [18]. This increased permeability is also considered to lead to a collapse of the MMP [19,20]. We demonstrated that photoactivated *Lonicera japonica* induced the opening of MPT pores accompanied by a disruption of MMP in CH27 cells. In addition, photoactivated *Lonicera japonica* induced a significant reduction in the capability for MitoTracker Red CMXRos uptake by mitochondria. These data suggested that mitochondria may be a target of photoactivated *Lonicera japonica* during photoactivated *Lonicera japonica*-induced CH27 cell death.

Molecular chaperones are major components of the cellular machinery involved in ensuring correct protein folding and the continued maintenance of protein structure. In this study, proteomic data showed that chaperones were involved in the photoactivated *Lonicera japonica*-induced CH27 cell apoptosis. DJ-1, HSP60 and HSP 70 are mitochondrial molecular chaperones. DJ-1 has been demonstrated to protect cells against oxidative stress and cell death [21]. Our results are also consistent with previous observations in which HSP60 and HSP70 play critical roles during apoptosis [7,8]. In the result of 2D gel electrophoresis, photoactivated *Lonicera japonica*-treated samples had a significant change in the expression of HSP70 protein (spot D4, #3 and #4). However, photoactivated *Lonicera japonica* had no effect on the protein expression of HSP70 by Western blotting analysis (Figure 3). HSP70 displays a spot family pattern in 2D gel. After cells were treated with *Lonicera japonica* extracts and irradiation, the protein amount of spot #3 and #4 significantly increased, but spot D4 decreased. In Western blotting data, the expression of HSP70 is the total HSP70 protein. Based on the above reasons, photoactivated *Lonicera japonica*-treated samples had a significant change in the expression of HSP70 protein in which the single spots of the complex pattern were probably due to post-translational modifications of one particular protein.

In this study, proteomic data also showed that photoactivated *Lonicera japonica* induced the change of endoplasmic reticulum molecular chaperone protein disulfide isomerase (PDI), which catalyzes the rearrangement of -S-S- bonds in proteins. It has been suggested that a variety of endoplasmic reticulum stresses result from unfolded protein accumulation, which induces the expression of molecular chaperones [22]. Therefore, we focused our attention on molecular chaperone expression and ER stress. This study demonstrated that the molecular chaperones in the endoplasmic reticulum, such as PDI, caspase-4 and 150 kDa oxygen-regulated protein (ORP150), are involved in photoactivated *Lonicera japonica*-induced CH27 cell apoptosis by increasing protein levels. It has been reported that caspase-4, an ER stress-specific caspase, is localized on the ER in human [23,24]. The 150 kDa oxygen regulated protein functions as a molecular chaperone in the endoplasmic reticulum [25]. Photoactivated *Lonicera japonica* also induced the changes of endoplasmic reticulum distribution, which was performed by an endoplasmic reticulum marker, in CH27 cells in this study. These results are consistent with previous observations that have shown an association between cell death and the increase in the ER stress which can induce compensatory responses [22,26]. Based on the data mentioned above, ER stress and molecular chaperones expression may play an important role in the photoactivated *Lonicera japonica*-induced CH27 cell apoptosis. It also seemed to indicate that the protein folding pathway might serve as an important apoptotic control point in photoactivated *Lonicera japonica*-induced apoptosis.

When cells accumulate ER stresses, ER chaperone proteins can be induced to alleviate protein aggregation and activate the proteosome machinery to degrade misfolded proteins. For survival, the cells induce endoplasmic reticulum chaperone proteins to alleviate protein aggregation, transiently attenuate translation and activate the proteosome machinery to degrade misfolded proteins. In the results of 2D electrophoresis, most of protein spots are more intense in control cell-loaded gels than in the photoactivated *Lonicera japonica*-treated cell-loaded gels in this study. Furthermore, the present study has demonstrated that the molecular chaperones in the endoplasmic reticulum, such as PDI and ORP150, and mitochondria, such as HSP60 and HSP70, are involved in photoactivated *Lonicera japonica*-induced CH27 cell apoptosis. These results seemed to indicate that heat shock proteins were synthesized by CH27 cells in response to *Lonicera japonica* and irradiation and a compensatory mechanism referred to as unfolded protein response (UPR) was triggered by photoactivated *Lonicera japonica*. It includes the inhibition of overall protein synthesis to decrease the protein-load, as well as the induction of endoplasmic reticulum chaperones and foldases, by which the cell attempts to increase the folding capacity in this study.

It has been suggested that excessive production of reactive oxygen species (ROS) may lead to oxidative stress, loss of cell function and ultimately apoptosis in cancer cells [27]. Haynes et al. (2004) also reported that prolonged activation of UPR, which normally functions to prevent ROS accumulation, resulted in oxidative stress and consequent cellular death [28]. Furthermore, reactive oxygen species producing is believed to play a key role in photosensitizer-induced photocytotoxicity. Many investigators have suggested that PDT involves the activation of a photosensitizer by irradiation with light in the presence of oxygen to produce singlet oxygen and other reactive oxygen species which cause cancer cells undergoing apoptosis or necrosis [14-17]. During the photodynamic process, photosensitizer generates a variety of ROS including singlet molecular oxygen (^1^O_2_, produced *via* type II mechanism) and superoxide anions (type I mechanism) [29,30]. In this study, treatment with 100 μg/ml *Lonicera japonica* for 2 h and 0.8 J/cm^2^ light dose resulted in significant increases in the intensity of the DCF signal as compared with those in the control. Therefore, the present study indicated that the production of ROS was involved in the photoactivated *Lonicera japonica*-induced CH27 cell death. Based on the above data, the study cannot verify that the amount of ROS was produced by photosensitizer *Lonicera japonica via* type I or type II mechanisms or induced by mitochondrial dysfunction and ER stress. However, the molecular chaperones should be triggered by photoactivated *Lonicera japonica* to counteract ROS accumulation during photoactivated *Lonicera japonica*-induced CH27 cell death.

## Conclusion

Our study applied 2D electrophoresis to analyze the proteins involved in photoactivated *Lonicera japonica*-induced apoptosis in CH27 cells. Photoactivated *Lonicera japonica* induces significant changes in the protein expression of chaperones in mitochondria and endoplasmic reticulum. Endoplasmic reticulum and mitochondria are the targets of photoactivated *Lonicera japonica* during photoactivated *Lonicera japonica*-induced CH27 cell apoptosis. The production of reactive oxygen species was demonstrated to be involved in photoactivated *Lonicera japonica*-induced CH27 cell apoptosis. In a comparative study among various fractions of the *Lonicera japonica* extracts, such as water, ethyl acetate and *n*-butanol fractions, the results showed that the ethyl acetate fraction had the highest photosensitizing activity in this study. This could explain the fact that the ethyl acetate fraction of *Lonicera japonica* extracts may contain compounds that exhibit the photosensitizing activity in CH27 cells. However, further studies are required to isolate and characterize the constituents which displayed photosensitizing activity present in ethyl acetate fraction of *Lonicera japonica* extracts.

## Abbreviations

BuOH: *n*-butanol; CHAPS: 3-[(3-cholamidopropyl)dimethylammonio]-1-propanesulfonate; CH27 cells: Human lung squamous carcinoma cell line; CM-H2DCFDA: 5-(and-6)-chloromethyl-20,70-dichlorodihydrofluorescein diacetate; DMEM: Dulbecco’s modified Eagle’s medium; DMSO: Dimethylsulfoxide; DTT: Dithiothreitol; EGTA: Ethylene glycol bis(b-aminoethylether)-N,N,N’,N’-tetraacetic acid; ER: Endoplasmic reticulum; EtOAc: Ethyl acetate; FBS: Fetal bovine serum; HSP: Heat shock protein; IEF: Isoelectric focusing; IgG: Immunoglobulin; JC-1: 5,5′,6,6′-tetrachloro-1,1′,3,3′-tetraethylbenzimidazolocarbocyanine iodide; MAPKs: Mitogen-activated protein kinases; MMP: Mitochondrial membrane potential; MPT: Mitochondrial permeability transition; MTT: 3-(4,5-dimethylthiazol-2-yl)-2,5-diphenyltetrazolium bromide; PAGE: Polyacrylamide gel electrophoresis; PBS: Phosphate-buffered saline; PDI: Protein disulfide isomerase; PDT: Photodynamic therapy; PMSF: Phenylmethylsulfonyl fluoride; ORP150: 150 kDa oxygen-regulated protein; ROS: Reactive oxygen species; SDS: Sodium dodecyl sulfate; UPR: Unfolded protein response.

## Competing interests

The authors declare that they have no competing interests.

## Authors’ contributions

LHZ designed the study, conducted the experiments and wrote the manuscript. LHZ, LJC and CWT helped to wrote the manuscript. LHZ, LJC, CWT, LYH and HMJ performed the experiments and analyzed the data. LJC and CWT equally contributed to this work. All authors read and approved the final manuscript.

## Pre-publication history

The pre-publication history for this paper can be accessed here:

http://www.biomedcentral.com/1472-6882/13/244/prepub

## References

[B1] Park HS Park KI Lee DH Kang SR Nagappan A Kim JA Kim EH Lee WS Shin SC Hah YS Kim GS Polyphenolic extract isolated from Korean *Lonicera japonica* Thunb. induce G2/M cell cycle arrest and apoptosis in HepG2 cells: involvements of PI3K/Akt and MAPKs Food Chem Toxicol 2012 50 2407 2416 10.1016/j.fct.2012.04.034 22561682

[B2] Xu Y Oliverson BG Simmons DL Trifunctional inhibition of COX-2 by extracts of *Lonicera japonica*: direct inhibition, transcriptional and post-transcriptional down regulation J Ethnopharmacol 2007 111 667 670 10.1016/j.jep.2007.01.017 17314019

[B3] Leung HW Hour MJ Chang WT Wu YC Lai MY Wang MY Lee HZ P38-associated pathway involvement in apoptosis induced by photodynamic therapy with *Lonicera japonica* in human lung squamous carcinoma CH27 cells Food Chem Toxicol 2008 46 3389 3400 10.1016/j.fct.2008.08.022 18796326

[B4] You BJ Wu YC Bao BY Wu CY Yang YW Chang YH Lee HZ Rottlerin Inhibits *Lonicera japonica*-Induced Photokilling in Human Lung Cancer Cells through Cytoskeleton-Related Signaling Cascade Evid Based Complement Alternat Med 2011 2011 193842 21331326 10.1155/2011/193842 PMC3038619

[B5] Dallmann K Junker H Balabanov S Zimmermann U Giebel J Walther R Human agmatinase is diminished in the clear cell type of renal cell carcinoma Int J Cancer 2004 108 342 347 10.1002/ijc.11459 14648699

[B6] van den Bogaerdt AJ El Ghalbzouri A Hensbergen PJ Reijnen L Verkerk M Kroon-Smits M Middelkoop E Ulrich MMW Differential expression of CRABP-II in fibroblasts derived from dermis and subcutaneous fat Biochem Biophys Res Commun 2004 315 428 433 10.1016/j.bbrc.2004.01.069 14766225

[B7] Lin KM Lin B Lian IY Mestril R Scheffler I Dillmann WH Combined and individual mitochondrial HSP60 and HSP10 expression in cardiac myocytes protects mitochondrial function and prevents apoptotic cell deaths induced by simulated ischemia-reoxygenation Circulation 2001 103 1787 1792 10.1161/01.CIR.103.13.1787 11282911

[B8] Beere HM Wolf BB Cain K Mosser DD Mahboubi A Kuwana T Tailor P Morimoto RI Cohen GM Green DR Heat-shock protein 70 inhibits apoptosis by preventing recruitment of procaspase-9 to the Apaf-1 apoptosome Nat Cell Biol 2000 2 469 475 10.1038/35019501 10934466

[B9] Garrido C Bruey JM Fromentin A Hammann A Arrigo AP Solary E HSP27 inhibits cytochrome c-dependent activation of procaspase-9 FASEB J 1999 13 2061 2070 10544189 10.1096/fasebj.13.14.2061

[B10] Feng R Zhai WL Yang HY Jin H Zhang QX Induction of ER stress protects gastric cancer cells against apoptosis induced by cisplatin and doxorubicin through activation of p38 MAPK Biochem Biophys Res Commun 2011 406 299 304 10.1016/j.bbrc.2011.02.036 21320468

[B11] Lee HZ Liu WZ Hsieh WT Tang FY Chung JG Leung HW Oxidative stress involvement in *Physalis angulata*-induced apoptosis in human oral cancer cells Food Chem Toxicol 2009 47 561 570 10.1016/j.fct.2008.12.013 19138722

[B12] Lindholm D Wootz H Korhonen L ER stress and neurodegenerative diseases Cell Death Differ 2006 13 385 392 10.1038/sj.cdd.4401778 16397584

[B13] Lemarie A Lagadic-Gossmann D Morzadec C Allain N Fardel O Vernhet L Cadmium induces caspase-independent apoptosis in liver Hep3B cells: role for calcium in signaling oxidative stress-related impairment of mitochondria and relocation of endonuclease G and apoptosis-inducing factor Free Radic Biol Med 2004 36 1517 1531 10.1016/j.freeradbiomed.2004.03.020 15182854

[B14] Chen R Huang Z Chen G Li Y Chen X Chen J Zeng H Kinetics and subcellular localization of 5-ALA-induced PpIX in DHL cells via two-photon excitation fluorescence microscopy Int J Oncol 2008 32 861 867 18360713

[B15] Donnelly RF McCarron PA Woolfson AD Derivatives of 5-aminolevulinic acid for photodynamic therapy Perspect Med Chem 2007 1 49 63 PMC2754918 19812736

[B16] Fotinos N Mikulic J Convert M Campo MA Piffaretti JC Gurny R Lange N 5-ALA derivative-mediated photoinactivation of Propionibacterium acnes J Dermatol Sci 2009 56 214 216 10.1016/j.jdermsci.2009.07.011 19709860

[B17] Sharma S Jajoo A Dube A 5-Aminolevulinic acid-induced protoporphyrin-IX accumulation and associated phototoxicity in macrophages and oral cancer cell lines J Photochem Photobiol B 2007 88 156 162 10.1016/j.jphotobiol.2007.07.005 17761434

[B18] Zoratti M Szabò I The mitochondrial permeability transition Biochim Biophys Acta 1995 1241 139 176 10.1016/0304-4157(95)00003-A 7640294

[B19] Crompton M The mitochondrial permeability transition pore and its role in cell death Biochem J 1999 341 233 249 10.1042/0264-6021:3410233 10393078 PMC1220352

[B20] Oliveira KA Zecchin KG Alberici LC Castilho RF Vercesi AE Simvastatin inducing PC3 prostate cancer cell necrosis mediated by calcineurin and mitochondrial dysfunction J Bioenerg Biomembr 2008 40 307 314 10.1007/s10863-008-9155-9 18679777

[B21] Takahashi-Niki K Niki T Iguchi-Ariga S Ariga H Function of DJ-1 in mitochondria Yakugaku Zasshi 2012 132 1105 1110 10.1248/yakushi.12-00220-3 23037695

[B22] Kudo T Okumura M Imaizumi K Araki W Morihara T Tanimukai H Kamagata E Tabuchi N Kimura R Kanayama D Fukumori A Tagami S Okochi M Kubo M Tanii H Tohyama M Tabira T Takeda M Altered localization of amyloid precursor protein under endoplasmic reticulum stress Biochem Biophys Res Commun 2006 344 525 530 10.1016/j.bbrc.2006.03.173 16630560

[B23] Hitomi J Katayama T Eguchi Y Kudo T Taniguchi M Koyama Y Manabe T Yamagishi S Bando Y Imaizumi K Tsujimoto Y Tohyama M Involvement of caspase-4 in endoplasmic reticulum stress-induced apoptosis and Abeta-induced cell death J Cell Biol 2004 165 347 356 10.1083/jcb.200310015 15123740 PMC2172196

[B24] Li J Xia X Ke Y Nie H Smith MA Zhu X Trichosanthin induced apoptosis in HL-60 cells via mitochondrial and endoplasmic reticulum stress signaling pathways Biochim Biophys Acta 2007 1770 1169 1180 10.1016/j.bbagen.2007.04.007 17570595

[B25] Ikeda J Kaneda S Kuwabara K Ogawa S Kobayashi T Matsumoto M Yura T Yanagi H Cloning and expression of cDNA encoding the human 150 kDa oxygen-regulated protein, ORP150 Biochem Biophys Res Commun 1997 230 94 99 10.1006/bbrc.1996.5890 9020069

[B26] Lai MY Hour MJ Leung WC Yang WH Lee HZ Chaperones are the target in aloe-emodin-induced human lung nonsmall carcinoma H460 cell apoptosis Eur J Pharmacol 2007 573 1 10 10.1016/j.ejphar.2007.06.061 17643413

[B27] Lau AT Wang Y Chiu JF Reactive oxygen species: current knowledge and applications in cancer research and therapeutic J Cell Biochem 2008 104 657 667 10.1002/jcb.21655 18172854

[B28] Haynes CM Titus EA Cooper AA Degradation of misfolded proteins prevents ER-derived oxidative stress and cell death Mol Cell 2004 15 767 776 10.1016/j.molcel.2004.08.025 15350220

[B29] Pass HI Photodynamic therapy in oncology: Mechanisms and clinical use J Natl Cancer Inst 1993 85 443 456 10.1093/jnci/85.6.443 8445672

[B30] Vath P Wamer WG Falvey DE Photochemistry and phototoxicity of aloe emodin Photochem Photobiol 2002 75 346 352 10.1562/0031-8655(2002)0750346PAPOAE2.0.CO2 12003123

